# Patient Activation through Counseling and Exercise – Acute Leukemia (PACE-AL) – a randomized controlled trial

**DOI:** 10.1186/1471-2407-13-446

**Published:** 2013-10-02

**Authors:** Mary Jarden, Tom Møller, Lars Kjeldsen, Henrik Birgens, Jesper Frank Christensen, Karl Bang Christensen, Finn Diderichsen, Carsten Hendriksen, Lis Adamsen

**Affiliations:** 1 The University Hospitals Centre for Health Research UCSF, Department 9701, Copenhagen University Hospital (Rigshospitalet), Blegdamsvej 9, Copenhagen, DK-2100, Denmark; 2 Center for Integrated Rehabilitation of Cancer Patients (CIRE), Copenhagen, Denmark; 3 Department of Hematology, Copenhagen University Hospital Rigshospitalet, Copenhagen, Denmark; 4 Department of Hematology, Herlev Hospital, Herlev, Denmark; 5 Department of Public Health, University of Copenhagen, Copenhagen, Denmark

**Keywords:** Acute leukemia, Cancer, Chemotherapy, Exercise, Health counseling, Physical and functional capacity, Quality of life, Symptoms, Outpatient management, Randomised controlled trial, Qualitative evaluation

## Abstract

**Background:**

Patients with acute leukemia experience a substantial symptom burden and are at risk of developing infections throughout the course of repeated cycles of intensive chemotherapy. Physical activity in recent years has been a strategy for rehabilitation in cancer patients to remedy disease and treatment related symptoms and side effects. To date, there are no clinical practice exercise guidelines for patients with acute leukemia undergoing induction and consolidation chemotherapy. A randomized controlled trial is needed to determine if patients with acute leukemia can benefit by a structured and supervised counseling and exercise program.

**Methods/design:**

This paper presents the study protocol: **P**atient **A**ctivation through **C**ounseling and **E**xercise – **A**cute **L**eukemia (PACE-AL) trial, a two center, randomized controlled trial of 70 patients with acute leukemia (35 patients/study arm) following induction chemotherapy in the outpatient setting. Eligible patients will be randomized to usual care or to the 12 week exercise and counseling program. The intervention includes 3 hours + 30 minutes per week of supervised and structured aerobic training (moderate to high intensity 70 - 80%) on an ergometer cycle, strength exercises using hand weights and relaxation exercise. Individual health counseling sessions include a self directed home walk program with a step counter. The primary endpoint is functional performance/exercise capacity (6 minute walk distance). The secondary endpoints are submaximal VO_2_ max test, sit to stand and bicep curl test, physical activity levels, patient reported outcomes (quality of life, anxiety and depression, symptom prevalence, intensity and interference). Evaluation of clinical outcomes will be explored including incidence of infection, hospitalization days, body mass index, time to recurrence and survival. Qualitative exploration of patients’ health behavior and experiences.

**Discussion:**

PACE-AL will provide evidence of the effect of exercise and health promotion counseling on functional and physical capacity, the symptom burden and quality of life in patients with acute leukemia during out patient management. The results will inform clinical practice exercise guidelines and rehabilitation programs for patients undergoing treatment for acute leukemia. Optimizing the treatment and care pathway may ease the transition for patients from illness to the resumption of everyday activities.

**Trial registration:**

ClinicalTrials.gov Identifier: NCT01404520.

## Background

Acute leukemia is a life threatening hematological malignancy, representing one percent of all cancers diagnosed annually in Denmark, which approximates to 200 new cases of acute myeloid leukemia (AML) and 50 acute lymphatic leukemia (ALL) cases in adults yearly (cancer.dk). The five-year international survival for AML is 40% for adult patients (<60 yrs) receiving curative intended treatment, and for ALL 50% (18–45 yr) and 10-30% (>45 yr) [1,2]. The WHO performance status score of patients with AML at the time of diagnosis has been shown to have prognostic importance [3]. The primary goal of treatment for acute leukemia is to rapidly achieve complete remission attained by the initial intensive chemotherapy regimen (induction), followed by repeated cycles of intensive chemotherapy (consolidation) and if indicated, an allogeneic stem cell transplantation. Each chemotherapy cycle is followed by severe neutropenia, thrombocytopenia and anemia, with an increased risk of serious infections, mainly septicemia and pneumonia [4]. Deterioration of nutritional status and quality of life (QOL) is reported after induction treatment [5]. Patients report a substantial symptom burden including lack of energy, shortness of breath, weakness, comprised physical functioning, difficulty sleeping, pain, nausea, emesis, diarrhea, weight loss, anxiety and distress [5-7]. Further, clinically significant depression can be precipitated or exacerbated by a diagnosis of acute leukemia [8]. Multiple symptoms have been found to have a deteriorating effect on QOL in patients with hematologic malignancy [9-11].

Over the past 15 years, patient safety aspects and prolonged hospitalization have been challenged by homecare interventions and outpatient management programs triggered by the prospect of improving QOL by reducing hospital admissions [12]. Implementation of outpatient programs for patients with acute leukemia have enabled patients to be discharged to their homes while receiving mandatory antibiotic prophylaxis during neutropenic phases [13,14]. For the patient, this requires frequent, lengthy, long term and supervised visits for treatment and follow-up care, ongoing transfusion support and close monitoring for complications including serious infections. Pharmacological strategies, as antibiotic prophylaxis, antiemetics etc. are frequently prescribed to improve tolerance to the planned chemotherapy treatment, and now physical activity in recent years has been used as a strategy for rehabilitation in cancer patients to remedy disease and treatment related symptoms and side effects. Evidence suggests that activation of cancer patients can result in healthy behaviors i.e. physical activity, and lead to better health-related outcomes and self care management [15,16]. In addition, tailored counseling sessions have been shown to improve functioning, clinical outcomes and reduce hospitalization [17]. Feasibility, safety and beneficial effects of low to high intensity exercise and psychosocial interventions have been found in cancer patients with the majority being breast cancer, and to a much lesser extent in colon, prostate, lung and hematological malignancies [18-21]. However, the complex clinical situation seen in acute leukemia has predominantly excluded patients from participating in exercise interventions. Furthermore, present clinical care practice does not incorporate health counseling and physical exercise during treatment for acute leukemia which leaves this particular patient group understudied and under recruited in existing exercise-based rehabilitation programs. In a recent review of exercise in hematological cancer survivors [22], only three small scale studies comprise patients with acute leukemia [23-25], and one pilot study with a mixed population of acute leukemia and lymphoma [26], all during hospitalization while undergoing the induction phase of chemotherapy treatment. These limited findings, indicate feasibility, safety and preliminary physiological and psychosocial benefits from exercise in patients during induction treatment for acute leukemia. In our recently published pilot study (n = 20), we intervened in the outpatient management setting by building a bridge between the hospital and daily life activities of patients with acute leukemia by addressing the challenges associated with maintaining muscle, cardiovascular and emotional and general health during chemotherapy treatment [9]. This 6 week supervised and structured exercise and health counseling intervention proved feasible, safe and well tolerated with physical, functional, psychosocial and symptom benefits in a small sample of patients with acute leukemia undergoing intensive chemotherapy. However, no recommendation for exercise can be issued specifically for acute leukemia patients undergoing induction and consolidation treatment during hospitalization and outpatient treatment and care [9,22].

This protocol paper presents our current randomized trial (PACE-AL) that aims to investigate the effect of a 12 week structured and supervised multimodal program of physical exercise and health counselling in patients with acute leukemia undergoing consolidation treatment during outpatient treatment and care. It is hypothesized that the intervention can minimize the loss of physical and functional capacity, reduce the symptom burden, improve psychosocial wellbeing and health related QOL. To our best knowledge, this is a first time study that examines a multimodal intervention of health counseling and exercise in patients with acute leukemia, initiated early in the treatment trajectory prior to consolidation treatment and conducted over a 12 week period. The long term effect of the intervention will be investigated at 6 and 12 month follow-up.

## Methods/design

### Participants and setting

This randomized controlled trial is a prospective, two group trial of a 12 week exercise and health counseling intervention in patients with newly diagnosed acute leukemia during the course of consolidation treatment in the outpatient clinic. 70 patients will be consecutively recruited and randomized by the research investigator and/or the nurse specialists from the Department of Hematology at two Copenhagen University hospitals: The University Hospital of Copenhagen, Rigshospitalet (RH) and Herlev Hospital (HH). Patients ≥ 18 years newly diagnosed with acute leukemia are eligible for the study upon completion of their initial induction chemotherapy with a documented complete remission (CR) status, able to read and understand Danish and can provide a signed informed written consent. The exclusion criteria are recent symptoms of cardiovascular, neurological or muscular disease, abnormal electrocardiogram and uncontrolled high blood pressure.

### Study and recruitment procedures

**Figure 1:**
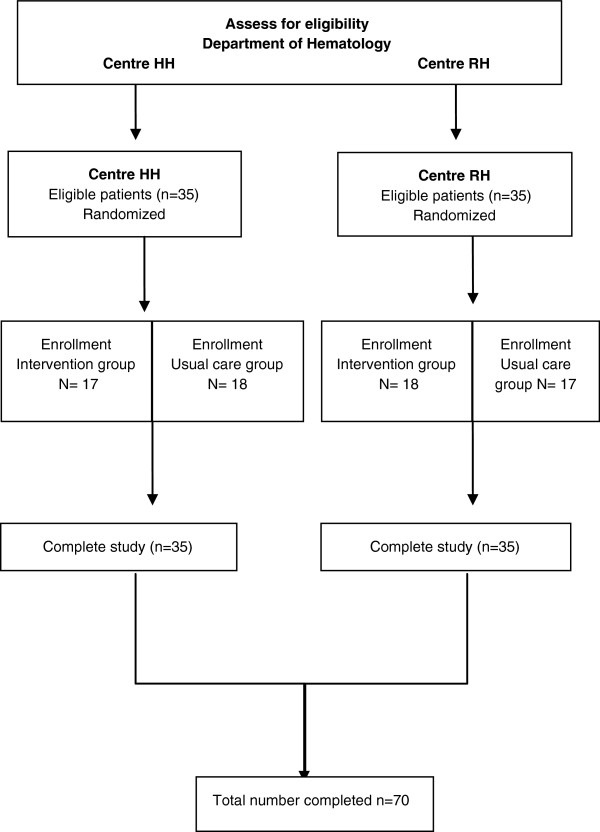
CONSORT diagram of patient recruitment and flow.

The study flow is presented in Figure 1. Potential participants will be identified and screened for eligibility by the research investigator and/or clinical nurse specialist by weekly reviews of patient admissions and their status through medical record review and consultation with the nursing and medical staff at the Departments of Hematology, RH and HH. If eligible, the patients will be approached once the initial induction chemotherapy treatment is completed and complete remission (CR) is achieved. The patients will be provided with verbal and written information regarding the study and subsequently asked if they would like to participate. Once written consent is obtained, the patients are immediately scheduled for their baseline tests, which include four physical/exercise tests and asked to complete patient reported outcome questionnaires (PRO). Baseline tests and assessments will be repeated at 6 weeks and at completion of the intervention period (12 weeks). Selected participants (purposive sampling strategy) will be interviewed at 12 weeks by the research investigator (n = 25). Further, PRO tests will be repeated at 6 and 12 months. Three brief PRO pertaining to symptom assessment and physical activity levels will be repeated once weekly throughout the duration of the study period. To strengthen internal validity, the procedures for tests/assessments will be conducted under the same or similar conditions at all three test points at both hospitals (HH and RH). The study personnel and blinded outcome assessors will conduct tests during the morning hours (between 9 am-12 noon), utilize the same test equipment and conduct the tests/assessments in a specific order.

### Randomization and group allocation

After baseline testing, patients will be randomized to one of two trial arms (intervention or control) using the computerized Clinical Trial Management System (CITMAS/HITMAS) [27] and stratified for age (<45 and >45 years), gender, and treatment facility (RH and HH). This is to ensure that the randomized groups are similar at baseline. A block design with allocation weight of 1:1 will be used to generate treatment allocation. Randomized patients will remain in the same group for the entire duration of the intervention.

### Blinding

Blinding the participants or the investigators to allocation group will not be possible, however the outcome assessors and the trials statistician will be blinded to the participants study allocation. The trials statistician will have access to unblinded data, but will prepare results without knowledge of allocation to randomization coding.

### Trial arm 1 (intervention protocol and general considerations)

The intervention program - *exercise and counseling* (Tables 1 and 2) was designed using principles for exercise suggested by the American College of Sports Medicine (ACSM) [28] and a literature review on physical activity and hematological cancer survivorship [22]. The counseling sessions and the daily guidance and motivation are theoretically based on the social cognitive model of health promotion [29], theory of planned behaviour [30] and motivational interviewing (MI) methods and principles [31-34]. The intervention is a supervised hospital-based program that takes place at designated training locations at two hematology out-patient departments (RH,HH). Each session is designed to last 1hour ±10 min., 3 days weekly during the patients out-patient visit between 10 am and 12 noon beginning the first day of ambulatory care and carried out for 12 weeks. Patients who are intermittently hospitalized due to infection or other complications during the study period will be offered the intervention in their hospital room and when possible, the patient will be asked to meet at the projects designated training areas. Consecutive inclusion will enable training in small groups between 2 and 4 participants.

**Table 1:** **The intervention program - *exercise and health promotion counseling***

**PHYSICAL ACTIVITY**			**COUNSELING**	
**Physical Training**	**Relaxation Training**	**Diet**	**Guidance and motivation**	**Health counseling**
*Supervised (out-patient facility at hospital)*				
Stationary cycling	Relaxation and breathing exercise with guidance and music	Protein/carbohydrate supplement 30 min. after training	Instruction and guidance in exercising, usage of step counter and walking program, short and long term motivation and symptom management	Focus:
Moderate to very hard intensity				1) Screening
				2) Health behavior
75-80% HR max	Low intensity	e.g. chocolate milk, shake, nuts, protein bar		2) Handling of symptoms
Interval training	RPE 6-9			3) Social/employment
RPE 15-17	MET 2.5			
MET 5.5 - 8		Protein: 16-19 g.		GOAL SETTING
		Carbohyd: 1400-1900 kj.		
Dynamic and resistance training			Individual	Individual
Moderate to hard				
2 sets, 12 reps				
RPE 14-16				
MET 5.5				
3 days/week				3 sessions 30-60 min.
*Not supervised (home, outdoors, hospital corridor etc.)*				
Walk program with step counter				
Daily and individual				

**Table 2:** **The weekly intervention program - *exercise and health promotion counseling***

**WEEKLY PROGRAM 12 wks., 3 days/wk., 3-4 hours/wk. and daily walking**				
**Monday**	**Tuesday**	**Wednesday**	**Thursday**	**Friday**
Training program incl. relaxation (1 hr.)		Training program incl. relaxation (1 hr.)		Training program incl. relaxation (1 hr.)
Protein snack		Protein snack		Protein snack
Guidance and motivation		Guidance and motivation		Guidance and motivation
step counter		step counter		step counter
**Week 1**	**Week 2 - 11**			**Week 12**
Walk program with step counter	Walk program with step counter			Walk program with step counter
Establish habitual level	Maintenance or progression			Maintenance or progression
**Week 6**				
(1) Health counseling	(2) Health counseling			(3) Health counseling
(30-60 min)	(30-60 min)			(30-60 min)

#### ***Intervention: pre-screening and safety precautions***

The research investigator and/or the study’s clinical nurse specialists and physiotherapists will pre-screen the patients and supervise all sessions. If one or more of the following criteria are present prior to the intervention, the session will be either postponed or modified on that specific day; diastolic blood pressure < 45 or > 95 mmHg, pulse at rest > 100/min, signs of infection (temp > 38°C); respiration frequency at rest > 20/min and signs of bleeding (petechiae, nose bleeds, bruises). The units’ transfusion policy is for thrombocytopenia, platelets < 15 × 10^9^/l and for anemia, hemoglobin < 7.5 - 9 g/dl. The patients will be continuously monitored with a digital heart rate monitor (Polar Model) and observed for adverse reactions/events (see Monitoring Adverse Events).

The supervised *exercise* component of the intervention comprises *stationary cycling*, and will be initiated for a period of 20-25 min for each session. A minimum of six work intervals are integrated to enable cardiovascular effect while ensuring safety and therefore aims to reach but not exceed 80% of the maximal heart rate calculated by ACSM formula (206.9 - (0.67 × age) + 75/80%) [35]. The subjective intensity of effort is evaluated by the Borg Rate of Perceived Exertion scale (RPE), a visual analogue scale ranging from 6 (light effort) to 20 (maximal exertion) [36]. Intensity effort will strive to be between 15-17 (hard to very hard). The metabolic equivalent intensity level (METS) is calculated for each physical activity component [37]. The main goal of aerobic progression is to gradually increase exercise intensity and duration. Six *dynamic and resistance exercises* using free hand weights will be carried out including bicep curl, shoulder press, squat, lunge, push-ups, and one core exercise for abdominal and back muscles. Exercises will be performed in 2 sets of 12 repetitions, and hand weights will be adjusted to enable 2 sets and up to 12 repetitions. Progression aims to increase weight. The exercises will be followed by *relaxation training* while lying supine (or sitting) on a mat following live instruction to music [38-40]. At completion of each training session, the patients will consume a single or combined protein/carbohydrate supplement (16-19 g. protein and 1600–2000 kj carbohydrate) within 30 minutes of exercise to speed recovery by repairing and rebuilding muscle tissue [41,42]. Training sessions will be accompanied by guidance and motivation by the research physiotherapists and clinical nurse specialists with special focus on topics related to the exercise program, short and long term motivation and symptom management [29-34].

*Counseling* will be conducted three times for 30-60 min. at baseline, six and twelve weeks. Health promotion counseling sessions are schematized and carried out by the research investigator and/or clinical nurse specialists through a circular process of pre-contemplation, contemplation, preparation, action and maintenance [43]. The main principle of health counseling is to create a partnership with the patient to improve adherence to the intervention during the study period and to motivate towards positive health behavior, including maintaining or increasing physical activity during and beyond the supervised exercise sessions, and also after completion of the program. MI is a behavioral change strategy that assumes the patient possesses inherent resources and motivation that may be enhanced through the patient’s own expression of goals [29-34]. Each counseling session is therefore goal oriented and offers guidance in aspects of managing treatment-related symptoms, including symptoms of stress and anxiety, and issues related to diet, smoking cessation, alcohol consumption, sleep, social and daily life function and physical activity.

At the start of the program, the participants will receive a step counter (Omron Walking Style Pro) with the motivational purpose of physical and behavioral awareness and activation [44]. The patients will be fitted with the step counter by the study physiotherapists and instructed to wear the step counter during waking hours. Results from the step counter will be electronically transferred to a laptop computer once a week for the purpose of reviewing with each patient the number of daily total steps and aerobic steps achieved during the previous week, which will result in the patient expressing physical activity and walking goals for the following week.

### Trial arm 2 (control group)

*The control group receives* standard care and treatment that does not include supervised physical activity and/or health promotion counseling at the departments. Patients in both the intervention and control groups will receive usual care and treatment, and are not restricted from participating in other physical activity during the study period.

### Study endpoints and assessments

Outcome measures will be assessed at three time points, baseline, six and twelve weeks with a 6 and 12 month follow up. Table 3 outlines the study assessment schedule. Demographic, performance status (WHO) and medical data will be collected by journal review and questionnaire. Assessments will be carried out between 9:00 am and 12:00 noon by the project outcome assessors at their respective hospitals. Patients will be instructed on proper technique for the tests, and will be advised to stop if they experience pain or extreme discomfort, nausea, or dizziness. During the test sessions, the patients will be continuously monitored with a digital heart rate monitor (Polar Model) and observed for adverse reactions/events (see Monitoring Adverse Events).

**Table 3:** Study assessment schedule

**Data Assessment**	**Screening**	**Day -2**	**Day 0**	**Day 1**	**6 weeks**	**12 weeks**	**6 months**	**12 months**
		**Baseline-test**			**Mid-test**	**Post-test**	**Follow-up**	**Follow-up**
*Recruitment*								
Chart review	x							
Patient approached	x							
Informed consent	x							
*Outcome Assessments*								
6MWD		x			x	x		
Aastrand-Rhyming test		x			x	x		
Sit to stand test		x			x	x		
Biceps arm curl		x			x	x		
Patient-reported outcomes		x			x	x	x	x
Medical chart review		x				x	x	x
*Weekly assessments*								
Step counter		x			x	x		
MDASI		x			x	x	x	x
BFI		x			x	x	x	x
PA Level		x			x	x	x	x
Randomization			x					
Intervention initiation				x				
Qualitative Interview						x		

**6MWD**, 6 minute walk distance; **MDASI**, M.D. Anderson Symptom Inventory; **BFI**, Brief Fatigue Inventory; **PA Level**, Physical Activity Level.

#### ***Primary endpoint***

*Functional performance/exercise capacity*: 6 minute walk distance (6MWD) which measures the sub maximal level of functional and exercise capacity and will be carried out in accordance with the American Thoracic Society (ATS) guidelines [45]. The ATS Pulmonary Function Standards Committee developed guidelines for the 6MWD in clinical settings [46]. The 6MWD was chosen because it is easy to administer in the outpatient setting, well tolerated by patients with acute leukemia, and reflects activities of daily living [47].

#### ***Secondary endpoints***

*Aerobic capacity.* A single stage 6 min submaximal exercise test; the Aastrand-Rhyming cycle ergometer test is used to predict VO_2_ max values [48]. The test is based on the linear relationship between VO_2_ and heart rate. The patients pulse will be continually monitored using a wireless heart rate transmitter. Resistance (Watt) is increased to elicit a steady-state heart rate between 125 and 170 beats/min at a speed between 60 and 65 r.p.m. If after 6 min the HR is above 125 beats/min and stable, not fluctuating more than 5 beats/min, the test is terminated. VO_2_ max is determined using a nomogram with an age and body weight correction factor. VOs max is stated in ml/kg/min.

*Functional performance and muscle strength* comprises three endpoints: *(1)* Sit to stand test will be performed as a measure of strength and performance of the lower extremity muscles. The 30 second sit to stand was developed as an assessment tool to measure lower body strength [49]; (2) Biceps Arm Curl measures arm flexor strength, upper body strength and endurance [50]. The aim is to perform as many arm curls as possible in 30 seconds; and (3) Measurement of weekly steps and distance with the step counter: Omron Walking Style Pro [44].

*Clinical outcomes* (exploratory endpoints) will include andropometric characteristics *(*body mass index (wt(kg)/ht(m^2^)), WHO performance status, hospitalization (days), increased temperature (>38°) (days), elevated C-reactive protein (days), infections and complications (type and days), neutropenia and thrombocytopenia (days), transfusions (platelets and RBC) (number), time to relapse and survival.

*Patient reported outcomes (PRO)* in this study are standardized and validated questionnaires that measure physical, functional, emotional and social wellbeing, symptom prevalence, intensity including fatigue and interference in daily activities. The PRO’s will be administered and checked for completeness by the study investigator at baseline, 6 and 12 weeks, and 6 and 12 months; and coded according to the guidelines given in the questionnaires manuals: (1) The Functional Assessment of Cancer Therapy-Anemia scale (FACT-An) assesses cancer specific Health Related Quality of Life (HRQOL), the fatigue symptom subscale (An) of the FACT-An scale and the Trial Outcome Index (TOI) [51]. (2) The 36-Item Short Form Health Survey (SF-36) assesses general wellbeing using eight scales measuring different aspects of general health with two summary scales; physical and mental component scales [52]. (3) The European Organization for Research and Treatment of Cancer Quality of Life Questionnaire (C30 EORTC QLQ-C30) assesses Quality of Life (QOL) using a global health status scale, five functional scales (physical, role, emotional, cognitive and social functioning) and symptom scales (fatigue, nausea and vomiting, pain, dyspnea, insomnia, appetite loss, constipation, diarrhea) [53,54]. (4) The Hospital Anxiety and Depression Scale (HADS) assess psychological wellbeing and is designed to measure general anxiety and depression in patients with physical illness [55]. (5) The M.D. Anderson Symptom Inventory (MDASI) assesses the severity of 13 symptoms and their impact as evaluated by six interference items [56]. The MDASI will be additionally administered once a week prior to the last training session. (6) The Brief Fatigue Inventory (BFI) assesses fatigue levels and their impact as evaluated by interference items [57]. The BFI will also be administered once a week prior to the last training session.

*Physical activity, social network/relations, activation and ability, and employment (return to work)* will be assessed by the (7) Leisure time physical activity [58], (8) Physical activity level [59], (9) Multidimensional Scale of Perceived Social Support (MSPSS) [60], (10) Patient Activation Measure (PAM) [61], (11) Self Efficacy Scale (GSE) [62] and (12) Employment status and ability [63].

*A qualitative in-depth face-to-face semistructured interview* (n = 25) at 12 weeks carried out by the study investigator will explore the patients’ perspectives on physical activity and health, health behavior, and the experiences and challenges (including barriers) of returning to daily life regarding issues of health (well-being, vitality/fatigue, strength, symptom control) and disease (treatment and management, symptoms, complications), social (network, family) and employment. Further, we would like to clarify whether and how the intervention has contributed to the patients’ experiences of physical, emotional and social wellbeing and adaptation. Moreover, we aim to describe the symptom experience from the patients’ perspective, as well as explore the effect of the intervention on the symptom burden and symptom interference. A topic and question guide will be used to aid the focus of the interviews.

#### ***Logbook***

A 'monitoring logbook’ will be used to document screening, adherence to the intervention, unwanted symptoms, adverse events, and self-reported symptom assessment and activity levels. All components of the intervention performed will be recorded on-going including; exercise mode, frequency, intensity, duration and progression as well as subjective exercise response (BORG) and heart rate. Components performed beyond that prescribed in the intervention program, i.e. weekends or evenings, will be reported by the patient and then documented. For each individual component in the programme, adherence to the intervention will be measured by calculating the percentage of recommended exercise sessions performed by the patient (number of sessions performed /number of sessions prescribed). The *control group* will receive a modified logbook and will be asked to register activity/exercise mode, frequency and duration [59], weekly, during the study period as well as register symptom prevalence, intensity and interference (weekly) using the symptom assessment scales MDASI [56] and BFI [57].

#### ***Tracking and monitoring adverse events***

Patients will be monitored for unwanted symptoms and adverse events during each intervention session and throughout the intervention period. The following study procedures will be incorporated to ensure safety: 1) Prior to each exercise session, the patient will be monitored for blood pressure, pulse, temperature and thrombocyte and hemoglobin levels according to the study’s screening criteria; 2) During each intervention session, the patients will be monitored for unwanted symptoms and adverse events and recorded in the patients study chart; 3) Symptoms experienced and adverse events will be discussed with individual patients weekly; 4) Adverse events will be a steady agenda at the weekly and monthly study group meetings; and 5) All adverse events will be reported by completing the hospitals unintended/accidental event form and will follow the hospitals procedure for reporting such events.

### Ethical considerations

There are presently no rehabilitation programs available for patients with acute leukemia at the Departments of Hematology (HH, RH), however upon approval from the physician, patients may be referred to a municipality rehabilitation program. Patients in both study arms are not inhibited from exercising during the study period, as long as it is in accordance with the departments’ safety guidelines for daily physical activity and social contact. Moreover, patients allocated to both the control and intervention groups will receive usual and optimal care during the study period.

#### ***Potential risks***

During the course of the trial, patients will receive two chemotherapy cycles and consequently experience two neutropenic periods, each lasting approx. 7–14 days and during this time patients are especially prone to experiencing unpleasant symptoms i.e. mucositis, fatigue, and complications i.e. bleeding and infections. Further, during the trial period, and as a requirement of usual care, patients will have an indwelling central venous catheter (CVC) placed in order to facilitate chemotherapy, transfusions and drug delivery. CVC’s have benefits (minimize extravasation risk and avoid discomfort), but are associated with certain risks (infection, thrombosis). Patients will therefore, be screened and observed (BP, P, T, platelets, hemoglobin, sign of infection or bleeding) prior to the physical tests and before and during each exercise session (see Pre-screening and safety precautions). We will integrate an adverse events procedure that includes observation, documentation, patient and study team awareness, discussion and reporting (see Tracking and monitoring adverse events). In our previous pilot study, no adverse events or injuries were observed as a result of the exercise [8], however two patients experienced aching in the hip area after increasing their walking distance and most patients reported slight soreness in their muscles the day after training. No bruising was noted. There were, however, two incidents during baseline testing of two patients, one female during the 6MWD tripped and fell to the floor, and a male patient fell forward and onto the floor during the sit-to-stand test. Both patients were not injured, and able to carry out the tests at a later time. Safety measures were then instituted immediately in the pilot study and these safety measures (using arm rests during the sit-to-stand test for weakened patients, patient information regarding wearing appropriate walking shoes) will continue during the RCT.

#### ***Approvals and registrations***

Study approval is obtained for Herlev Hospital and Rigshospitalet by the Scientific Ethics Review Committee of the Capital Region of Denmark (J.no. H-4-2010-046) and the Danish Data Protection Agency (J.no. 30–0431). The study is registered at ClinicalTrials.gov Identifier: NCT01404520.

### Statistical considerations

#### ***Sample size***

This randomized controlled trial will include 70 acute leukemia patients over a recruitment period of 34–36 months. Sample size calculation is based on the primary endpoint; the 6 min walk distance (6MWD) and the results from our pilot study (n = 20), where n = 17 completed the study (15% lost to followup). The pilot study baseline for 6MWD was 450 m and the average change was 56.3 m effect (SD = 69.2). Assuming that there is no change in the control group, an inclusion of 70 patients (35 intervention, 35 control) will yield more than 90% power (risk of type 2 error set at 0.10) to detect a difference of this magnitude between groups using a significance level of 0.05 (risk of type 1 error set at 0.05). Should 20% be lost to followup (n = 56), the power will still be larger than 80%.

### Analytic plan

#### ***Quantitative data***

Data will be entered into the OpenClinica database [64] and statistical analysis will be carried out using Statistical Analysis Systems (SAS) version 9.2. Procedures for data entry and audit program have been developed to ensure accurate data entry [65]. *Baseline comparisons* of demographic and clinical outcomes will be performed using independent samples t-tests, and chi-squared tests will be applied for categorical variables. The *primary endpoint* will be reported as an independent samples t-test comparing change scores in the two randomization groups. The *secondary endpoints* (physical and strength tests and PRO questionnaires) will be reported as means and 95% confidence intervals and medians inter quartile range (IQR) for continuous variable, while categorical data will be reported as proportions and compared across randomization groups using chi-squared tests. The intent-to-treat principle will be applied and the significance level is set at 0.05. *Additional analyses* of 6 and 12 month followup data (PRO questionnaires) and of weekly symptom measurements and physical activity data will utilize linear mixed models to quantify trajectories. The trials statistician (KBC) will prepare results without knowledge of assignment to randomization coding.

#### ***Qualitative data***

Upon consent from the participants, the interviews will be recorded and transcribed verbatim. Data will be anonymised and coded using computerized qualitative data analysis software (NVivo 10) [66]. Thematic analysis is a qualitative analytic method that identifies, analyzes and reports themes and patterns within and across cases [67,68]. This study will apply an inductive, data-driven approach, which is the process of coding the data without attempting to fit it into a preexisting coding frame. The six phases of thematic analysis are as follows: (1) First, the investigator listens to the audio recordings and reads/re-reads the transcripts to become familiar with the content of the data. (2) Next, initial codes are generated from data extracts (sentences or paragraphs), (3) and from these extracts, the investigator searches for and reviews the themes, (4) generates a thematic map of the analysis, (5) defines and labels themes and finally, (6) identifies common themes. This final phase is the analysis of selected extracts/themes in relation to the initial research questions, literature and theory [67,68].

## Discussion

PACE-AL will target the special clinical and rehabilitation needs of patients with acute leukemia. Several aspects were taken into consideration in the development of PACE-AL to assure safety and tolerability, adherence and completion of study requirements. Firstly, the timing of initiation of the intervention along the treatment trajectory was considered. Once the diagnosis is established, patients are immediately hospitalized and induction chemotherapy with curative intent is rapidly initiated. The patients’ medical situation at this time is unstable with a particular risk of severe infectious complications [69,70]. Additionally, it can be a challenge for patients to face the uncertainty of the disease and treatment while experiencing treatment-related symptoms and side effects. Being that safety is our main concern, we chose to recruit patients and initiate the intervention after completion of induction treatment, and once the patients’ condition is stabilized (CR status). After induction treatment, patients are typically managed as outpatients for months and it is during this phase in the treatment trajectory that PACE-AL trial will be initiated. Secondly, it is reported that patients with hematological malignancies undergoing intensive chemotherapy have a low level of 'naturally occurring’ physical activity, suggesting that a structured intervention may be necessary in order to promote exercise in this population [71]. However, there are no exercise guidelines available for patients with acute leukemia. Most exercise trials and rehabilitation programs institute screening parameters for participation that require platelet levels of at least 40-50 x 10^9^/l. The units’ transfusion policy (HH, RH) for thrombocytopenia is platelets < 15 × 10^9^/l, which on a daily basis and without platelet transfusion would exclude patients in this group from participation in existing programs. A few trials have safely carried out moderate physical exercise in patients undergoing intensive chemotherapy with platelet values ≥20 × 10^9^/l [22,72-74] and >10 × 10^9^/l [26]. PACE-AL will allow exercise participation with platelet values ≥15 × 10^9^/l. [9] The safety precautions instituted in this trial include screening (platelet level, temperature, blood pressure and signs of infection and bleeding) prior to the physical tests and intervention, supervision and monitoring during the intervention, as well as a procedure for monitoring and reporting intervention-related adverse events. Thirdly, we considered the treatment setting. We chose to offer the intervention during the patients waiting time at the outpatient department to avoid prolonging the time spent at the hospital. Further, knowing that patients have difficulty initiating independent exercise during intensive chemotherapy in this setting, we decided to lengthen the intervention period from 6 weeks in our pilot study [9] to span over two chemotherapy cycles (12 weeks). The fourth aspect is the intervention type and dose. Since patients during outpatient management are required to visit the unit 3–4 days/week, an exercise intervention of 3 days/week would be realistic for the patient. Moreover, we anticipate that patients will be experiencing a relatively high symptom burden and in effect, do not want to hold the patient longer than necessary at the department. Therefore, each session will not exceed 1 hr ± 10 min. We designed the components of the intervention based on general guidelines suggested by the ACSM [28] and a literature review on physical activity and hematological cancer survivorship [22]. The exercise components in this study will include a combination of aerobic, resistance and relaxation training of low to moderate intensity 3 days/week á 3–4 hours/week. The walking program with step counter will be applied as motivation for independent activity. The step counter will be used as a means of building a bridge between hospital and daily life to improve the naturally occurring level of physical activity while at home. The step counter enables the study team to electronically review the data, and give the patient feedback about their independent activity level outside the hospital setting. Knowing that patients are at risk of losing muscle mass during treatment due to inactivity, prednisolone treatment etc., we will incorporate protein/carbohydrate supplements after each training session to prevent muscle wasting. Finally, the health counseling and daily motivation and guidance are important components in this intervention. Exercise trials for patients with hematological malignancies are primarily designed as exercise-only interventions and do not include psychosocial interventions or report the efforts made to guide and motivate participants during the individual sessions, across the intervention study period and long term [22,25,75].

We chose validated physical and functional capacity tests that would be feasible in this acute leukemia clinical setting. The tests reflect physical, functional and aerobic capacity and upper and lower body muscle strength. The 6MWD was chosen for the primary endpoint because it reflects activities of daily living, is easy to administer in the outpatient department and well tolerated by patients with acute leukemia undergoing chemotherapy. The gold standard of maximal oxygen uptake requires patients to perform a maximal stress test, however, wearing a mask is uncomfortable for patients recovering from stomatitis, as reported by Dimeo [76]. Therefore, oxygen uptake in this study as a secondary endpoint will be estimated by a submaximal cycle test, requiring a work intensity that will be realistic for weakened patients at all test points [48].

## Summary

Inactive periods during treatment for acute leukemia are frequently related to fatigue and depression, and can lead to reduced cardio respiratory capacity, as well as impaired muscular function, affecting the ability to carry out activities of daily living. In order to optimize the treatment and care pathway, appropriate exercise guidelines and rehabilitation programs for patients undergoing treatment for acute leukemia need to be established to ease the transition from illness to the resumption of everyday activities, e.g. job/school. PACE-AL trial will study the effect of a 12 week supervised and structured exercise and health promotion counseling intervention on physical and functional capacity, symptom burden and symptom interference with daily activities, quality of life, psychosocial and clinical outcomes in patients with acute leukemia undergoing intensive chemotherapy during outpatient management.

## Abbreviations

PACE-AL: Patient Activation through Exercise and Counseling – Acute Leukemia; CR: Complete remission; ACSM: American College of Sports Medicine; 6MWD: 6 minute walk distance; FACT-An: Functional assessment of cancer therapy-anaemia scale; SF36: 36-item short form health survey; EORTC QLQ-C30: The European Organization of Research and Treatment of Cancer Quality of Life Questionnaire; MDASI: M.D. Anderson symptom inventory; BFI: Brief fatigue inventory; PA Level: Physical activity level; Leisure Time PA: Leisure time physical activity; MSPSS: Multidimensional scale of perceived social support; PAM: Patient activation measure; GSE: General self efficacy; IQR: Inter quartile range; HH: Herlev Hospital; RH: Rigshospitalet; UCSF: The University Hospital’s Centre for Health Research; CIRE: Center for Integrated Rehabilitation of Cancer Patients.

## Competing interests

The authors declare that they have no competing interests.

## Authors’ contributions

MJ devised the study concept and design and drafted the manuscript. LK, HB, FD and CH participated in the design of the study and revised the manuscript for important intellectual content, JFC contributed to the design of the exercise components in the intervention, KBC conducted the power calculation, devised the analytic/statistical plan and revised the manuscript for important intellectual content. TM and LA contributed to the conception and design of the study, and revised the manuscript for important intellectual content. All authors read and approved the final manuscript.

## Pre-publication history

The pre-publication history for this paper can be accessed here:

http://www.biomedcentral.com/1471-2407/13/446/prepub
